# Advances in Molecular and Genetic Technologies and the Problems Related to Their Application in Personalized Medicine

**DOI:** 10.3390/jpm14060555

**Published:** 2024-05-23

**Authors:** Valeriya Nakhod, Anton Krivenko, Tatiana Butkova, Kristina Malsagova, Anna Kaysheva

**Affiliations:** Institute of Biomedical Chemistry, 10 bld. 8, Pogodinskaya str., 119121 Moscow, Russia

**Keywords:** molecular and genetic technologies, personalized medicine, biotechnology, microarray

## Abstract

Advances in the global personalized medicine market are directly related to innovations and developments in molecular and genetic technologies. This review focuses on the key trends in the development of these technologies in the healthcare sector. The existing global developments having an impact on the evolution of the personalized medicine market are reviewed. Efficient measures to support the development of molecular and genetic technologies are proposed.

## 1. Introduction

Today, the global biotechnology market and its evolution constitute an important segment in the healthcare industry. Biotechnology is a field of research and innovation that combines the biological and technological principles for creating products and services aiming to improve quality of human life and solve many medical issues.

It was not until several years ago that biotechnology started to be viewed through the prism of the financial sector, currently being among the highest-potential market segments. In 2022, during the 26th Global Congress on Biotechnology, it was noted in a market analysis report that the global biotechnology market size was valued at USD 1023.92 billion in 2021 and was poised to grow during the period between 2023 and 2030 at a compound annual growth rate (CAGR) of 13.9% [1]. Government activities aimed at modernizing the legal and regulatory framework, improving the regulatory approval process and reimbursement practices, as well as standardizing clinical trials, contribute to the market boom. The growing impact of personalized medicine and the increasing number of orphan drug formulations open up new avenues for using biotechnology and engaging new creative biotechnology companies, which helps increase market revenue.

According to the analytical market research company Research and Markets, the global biotechnology market size is expected to reach USD 3.88 trillion by 2030 [2].

Modern biotechnologies are widely used in various production processes in the agri-food, environmental, industrial, and healthcare sectors. The area attracting researchers the most is healthcare, and the pharmaceutical industry in particular, since it is directly related to increasing the population’s life expectancy, unlike other sectors.

One must bear in mind that biotechnology, especially in the context of personalized medicine, has a great potential for transforming a country’s economy:

1. Investment in biotechnology related to personalized medicine catalyzes research and development. Therefore, novel technologies as well as diagnosis and treatment methods are created, in turn contributing to growth of the industry and the economy as a whole.
2. Application of biotechnologies in personalized medicine allows one to develop customized treatment approaches taking into account patients’ genetic characteristics. It reduces the cost of treating complications and improves healthcare outcomes, eventually saving resources and increasing efficiency.
3. Furthermore, advances in this sector will also contribute to creating new jobs due to the founding of new companies and laboratories as well as launching innovative projects.
4. Countries investing in biotechnology and personalized medicine solidify their global market position. The cutting-edge medical technologies and chances to receive personalized treatment are attractive for patients, which spurs the development of medical tourism and attracts more foreign patients and medical experts to the country.

## 2. Methods

The main research method was analyzing the information sources showing the state of the art for the topic being discussed. The information sources included peer-reviewed specialized academic journals and open-source information systems.

To conduct this review, we conducted an extensive literature search across multiple databases (PubMed, Scopus, and Web of Science) to identify relevant studies published up to the end of 2023 (Table 1). The review authors held collaborative discussions to consolidate their findings and prepare the current narrative review.

## 3. Results

### 3.1. Development Trends for Molecular and Genetic Technologies

According to a study conducted by Global Market Insight, the biotechnology market size is expected to reach over USD 729 billion by 2025 and will grow at a high pace due to the increasing prevalence of chronic diseases [3].

In the long run, the global challenges for the biotechnology market include development of the following spheres [4]:

- pharmaceuticals: vaccine formulations, personalized drugs, identification of novel molecular biomarkers, and molecular diagnostics;
- personalized medicine: microarrays and biosensors;
- agrobiotechnology: genetic engineering, biologically pure foods, etc.;
- bioindustry: DNA sequencing, recombinant technologies, fermentation, tissue engineering, chromatography, PCR technologies, nanobiotechnology, and cellular analysis;
- bioinformatics: elaborating algorithms for big data storage and management.

The results of recent research offer a wide range of opportunities for improving quality of human life. The potential is not confined to treatment of pathological conditions: new avenues are opening up in such areas as regenerative, personalized, and preventive medicine, as well as the feasibility of tissue and organ repair, which will undoubtedly have a favorable effect on life expectancy and active longevity.

The conventional DNA sequencing methods were developed in the late 20th century and are currently used very commonly in research and medical diagnostics institutions. It took 13 years to sequence a human genome under the Human Genome Project. This branch of biomedicine has been evolving over the past 5–6 years, and more than 100,000 human genomes have been sequenced [5]. The Illumina HiSeq^®^ system can currently generate 200–600 gigabytes of sequence data per cycle. The cost of whole-genome sequencing has dropped dramatically from USD 14 million (2006) to USD 1500 (2016). The Veritas Genetics company (Danvers, MA, USA) now offers standard whole-genome sequencing for USD 599.

Advances in the DNA microarray technology [6], first-generation Sanger sequencing [7], next-generation sequencing (NGS) [8], and third-generation sequencing (TGS) [9] have enabled deep whole-genome/exome sequencing to characterize the mutational landscape of the analyzed samples.

There are two types of DNA microarrays: single- and two-channel ones, e.g., DNA microarrays manufactured by Agilent Santa Clara, CA, USA) [10] or Affymetrix GeneChip (Santa Clara, CA, USA) [11].

There are four next-generation sequencing (NGS) strategies:

- cyclic-array sequencing [12];
- microelectrophoretic methods [13];
- sequencing by hybridization (SBH) [14];
- single-molecule real-time sequencing [15].

These technological opportunities have greatly improved the sequencing speed and scalability. NGS is superior to Sanger sequencing in terms of speed, throughput, genomic library construction, and cost (savings being USD 30 to USD 1249 per patient in diagnosing cancer) [16]. However, NGS has a number of limitations:

- short read length (the average read length ranges from 32 to 330 base pairs) and
- low accuracy compared to that for Sanger sequencing.

Moreover, the cost of genome sequencing is a relevant issue, since it still ranges from USD 1 to USD 60 per megabase, although it has already declined by several orders of magnitude compared to the cost of Sanger sequencing.

Third-generation sequencing (TGS) is a unified molecular technology that allows real-time long-read sequencing (from one thousand to millions of nucleotides) with minimal errors.

Despite the rapid development and growing reputation of NGS and TGS, DNA microarray technology is still a popular tool in genome-wide association studies (GWASs) for reducing costs, and combining DNA microarrays with NGS/TGS in genotyping to increase the resolution of population-specific haplotypes as well as research validity is becoming increasingly common [17,18,19].

Genome sequencing technologies have been used to assess many genetic disorders, such as:

- high-identity segmental duplications [20];
- diseases associated with BRCA1/2 gene mutations [21];
- sequence variations [22];

and to explore the genomic landscape of complex diseases such as endometriosis [23] and thyroid cancer (Iqbal et al., 2022), as well as detect novel alleles of polymorphic gene clusters [24,25].

The size of the global DNA sequencing market is expected to reach approximately USD 37.99 billion by 2032 vs. USD 9.69 billion in 2022, the compound annual growth rate (CAGR) being 14.7% during the 2023–2032 forecast period [26].

This period has also been witnessing a boost in transcriptome analysis technologies. Serial analysis of gene expression was one of the first methods used for identifying messenger RNA transcripts in large quantities [27]; however, it has given way to the major modern transcriptomics technologies: microarrays and the RNA-Seq technique. Serial analysis of gene expression allows one to analyze up to one thousand transcripts, whereas the next-generation sequencing methods (RNA-Seq) have made it possible to analyze hundreds of thousands of transcripts belonging to different RNA classes.

Mass spectrometry is the predominant technology being developed in the field of proteomics. Application of the electrospray ionization technique has become one of the key advances [28,29,30]. These studies enabled quantification of several hundred proteins. Today, several thousand proteins can be quantified by mass spectrometry.

Like genomics, transcriptomics, and proteomics, metabolomics has been largely driven by technological advances: the development of high-performance mass spectrometry has contributed to the evolution of metabolomics as an “omics” discipline. Unlike proteomic analysis, specific sample preparation protocols need to be elaborated for metabolomic analysis because metabolites have a broad range of physicochemical properties [31]. Liquid chromatography–mass spectrometry and gas chromatography–mass spectrometry are preferentially used in metabolomics to overcome this problem.

Metabolomics has also benefited from the development of nuclear magnetic resonance techniques, which had originally been elaborated for a different purpose (namely, to characterize the 3D structure of a protein). Each of these analytical platforms has its own advantages and shortcomings [32]. Nuclear magnetic resonance spectrometers provide reproducible results, allow one to perform quantitative metabolite identification, but are characterized by low sensitivity. Mass spectrometry coupled with liquid and gas chromatography ensures high analysis sensitivity and reliable metabolite identification. However, the preliminary sample preparation procedures (extraction and derivatization) are time- and labor-consuming.

The metabolomics market size was valued at USD 1.84 billion in 2020 and is expected to reach USD 4.95 billion by 2028, growing at a CAGR of 13.13% over the period between 2021 and 2028. The rise in global geriatric population size, as well as the prevalence of cancer, neurodegenerative diseases (including Alzheimer’s disease), endocrine diseases, and diabetes mellitus in particular, are among the factors spurring the growth of this market [33].

**Figure 1:**
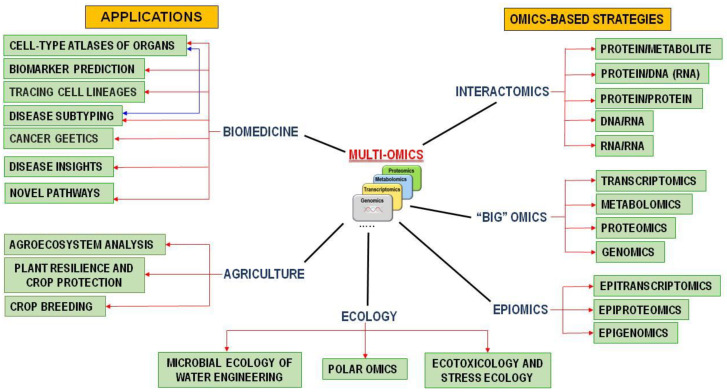
Multiomics approaches and some areas of practical application.

There are several trends in the development of the “omics” technologies. The first one consists in solving the technical problems (designing new ionization sources and ion detectors). Another trend is discovering the novel types of “omics”. With greater researchers’ understanding of the importance of cellular homeostasis at various organization levels with respect to treatment of human diseases, there emerged a tendency towards systematic investigation of a particular area of knowledge through integration of “omics” data. Novel “omics”, such as immunomics, translatomics, microbiomics, interactomics, and redoxomics, have recently emerged. Redoxomics is a novel type of omics based on knowledge about the role of redox homeostasis in maintaining the healthy state of cells and in the pathogenesis of various diseases (Figure 1).

### 3.2. Developments Contributing to the Progress in Personalized Medicine

Gene, cell, and RNA therapies are currently viewed as a single combination of technologies based on which various types of products (cell-based drugs or pharmaceuticals, vaccines, etc.) are being developed.

The potential range of microarray applications is rather broad, encompassing the following areas:

- oncology: diagnosing a large number of diseases at the early (preclinical) stage;
- allergology: screening across 17 mixed allergen groups (house dust, grass and tree pollen, pets, etc.), as well as inhalant and food allergens;
- cardiology: rapid diagnosis of cardiovascular diseases outside of specialized medical settings;
- infectious diseases: early symptoms of some infectious diseases are often almost identical, while their therapies differ fundamentally, so efficient segmentation of such diseases is a very relevant area;
- dentistry: microbiological analysis of the oral cavity to detect various types of bacteria;
- transfusion medicine: in the near future, laboratory screening for all the parameters (blood group, serology, bacteriology and virology testing) in blood biorepositories will be based on the DNA microarray technology [34];
- pharmacotherapy: assessment of the effectiveness of personalized therapy and the risk of adverse reactions, toxicity testing of new drugs and cosmetic products during the development stage, which will exclude laboratory animals from the development process (Table 2) [34].

Despite the abundant data on biomarker detection, no tools are currently available for integrating the results into practical medicine. Furthermore, the lack of standardized tools, methods, and a well-ordered process for approving several markers hinders the launching of novel biomarkers into clinical practice for patients having different diseases.

Advances in the cutting-edge technologies characterized by superior analytical performance and low cost, such as the programmable bio-nanochip system, can transform the healthcare service delivery. Well-planned device design as well as development and distribution plans enable the implementation of scientific discoveries in such fields as genomics, proteomics, metabolomics, and glycomics by converting the information within the key biomarkers into signatures that will enable the use of personalized treatment options for physicians and patients. Integration of these new platforms with mobile health (mHealth) applications may become demanded, since 70% of all medical decisions regarding patient diagnosis, treatment, hospitalization, and discharge are made based on the results of laboratory tests and instrumental examinations.

Since healthcare activity focuses on early diagnosis, timely and accurate laboratory results from mobile health applications can ensure better patient care and effective cost management [44].

Health status depends 80% on one’s lifestyle, habits, environmental quality, and diet, while genetic factors account for the remaining 20%. Today, biotechnological tools can be used to correct almost all environmental factors in order to preserve health and improve quality of life. The current knowledge about the human genome allows one both to identify risks associated with genetic factors and to manage them.

Several commercial products, such as 454 sequencers (Roche Life Science, Branford, CT, USA), Illumina’s genome analyzers (Illumina, San Diego, CA, USA) [45], and the SOLiD platform (Applied Biosystems, Foster City, CA, USA), were widely used for next-generation sequencing (NGS) and have made significant contribution to the “omics” technologies. However, the Roche 454 sequencers and the SOLiD platform were later withdrawn from the market, so Illumina remained the only company dominating the field. After buying Complete Genomics, the Beijing Institute of Genomics has entered the sequencing market to become a progressive institution that could design sequencers. Other NGS platforms, such as Ion Torrent (Thermo Fisher, Waltham, MA, USA), also hold a certain market share.

The first Russian genome in a bottle (GIAB) aiming to characterize human reference genomes was announced at the conference “2022 Genome Sequencing and Editing”. Today, the American NA12878 is the most popular “genome in a bottle”. There are also Asian analogs: YH. The Russian standard genome is currently being developed. It has been denoted as E701. No cell line has been generated for maintaining the genome yet, but there is a test tube containing the genomic DNA deposited at −80 °C. Whole-genome and whole-exome sequencing have been performed thus far, and a file integrating the resulting data has been compiled.

The mass spectrometry market is highly competitive. The key stakeholders offer a wide range of mass spectrometry products and have a broad geographical scope. Companies such as Agilent Technologies (Santa Clara, CA, USA), Bruker Corporation (Billerica, MA, USA), Danaher Corporation (Columbia, WA, USA), Leco Corporation (Saint Joseph, MI, USA), Perkin Elmer Inc. (Waltham, MA, USA), Shimadzu Corporation (Kyoto, Kyoto, Japan), Thermo Fisher Scientific (Waltham, MA, USA), Waters Corporation (Milford, MA, USA), SCIEX AB (Framingham, MA, USA), Analytik Jena (Jena, Germany), JEOL Ltd. (Akishima, Tokyo, Japan), Hiden Analytical (Warrington, UK), and MKS Instruments (Andover, MA, USA) hold a significant share of the mass spectrometry market.

Russia’s first serial quadrupole mass spectrometric (mass-selective) detector, a chromatography/mass spectrometry system, has been designed by engineers of the Special Design and Technological Bureau “Chromatec”. In 2022, the Skolkovo Innovation Center presented a prototype of mass spectrometer that allows one to conduct research in various fields such as biology, pharmaceutics, medicine, and nuclear energy. Alpha LLC (St. Petersburg, Russia) is a manufacturer of MX5313 chromatography–mass spectrometers aiming to measure the contents of the components of organic and inorganic mixtures in compliance with approved standardized methods.

The revenue of the global mass spectrometry market was estimated at USD 5.4 billion in 2023 and is expected to reach USD 7.8 billion by 2028, the average CAGR for the 2023–2028 period being 7.5% [46].

North America holds the largest share of the global mass spectrometry market. There is growing funding for research and government initiatives in this region, as well as the widespread use of mass spectrometry in metabolomics and the oil industry.

Several projects have been launched in the segment of specialized services. Thus, the National Bioservice LLC holds the key position among biobanks. The Novel Software Systems company, with their Genomenal platform, is cultivating the market of processing and interpreting the human DNA data obtained using eight different models of sequencers and microarrays.

### 3.3. Nanomedicine—Medical Applications of Nanotechnology

Developments in the realm of nanotechnology are closely related to the development and needs of personalized medicine. Nanotechnology in medicine is the use of nanoscale materials and devices to diagnose, treat, and monitor diseases. Nanomedicines have unique properties such as high surface activity and specific interaction with biological structures, which makes them ideal candidates for elaborating state-of-the-art methods of medical intervention. Application of nanotechnology includes the development of nanoparticles for drug delivery, construction of nanodiagnostic tools for early recognition of diseases, and development of nanobiosensors for health monitoring. These technologies represent great opportunity for personalized medicine and allow tailoring of a patient’s therapy to provide more effective and safe treatment (Table 3).

Compared to conventional drugs, nanomedicine offers a number of physical and biological advantages. These include improved solubility and pharmacokinetics, increased efficacy, reduced toxicity, and enhanced tissue selectivity. In 2017, there were more than 50 nanopharmaceuticals that received FDA approval and were available for clinical use [57]. Among nanomedicines, the largest share is occupied by drugs for the treatment of cancer (53%) and infections (14%). Other developments have occurred in blood diseases, endocrine and metabolic diseases, diseases of the nervous system, immunological and cardiovascular diseases, and eye diseases [58]. In addition, nanomedicines are used in vaccine development and diagnostic imaging. Nanomedicine pharmaceuticals are classified in liposomes or lipid-based nanoparticles (33%), antibody–drug conjugates (15%), polymer-drug/protein conjugates (10%), and polymer nanoparticles (10%) [59].

Health monitoring and early diagnosis represent enormous potential for the development of personalized medicine and cover the following aspects:

- highly sensitive biosensors and nanoparticles with a functionalized surface for phishing and recognition of biomarkers in the body for early diagnosis of diseases [60];
- nanoparticles for delivering drugs directly to affected tissues or organs in order to increase the effectiveness of treatment, as well as reduce negative side effects [61];
- nanodevices for monitoring various health indicators (pulse, glucose and oxygen levels in the blood) in real time. The collected data can be transferred to a smartphone or other device to warn of possible problems [62];
- nanomaterials for the needs of regenerative medicine, used in the creation of biocompatible materials [63].

For a long time, centers for personalized medicine have been extensively involved in research and development of innovative approaches, including nanotechnology-based ones, which are focused on the treatment of various diseases. Such medical centers combine expertise in genomics, proteomics, bioinformatics, and pharmacology and that of attending physicians. For example, the Research Center in Nanomedicine and Pharmaceutical Nanotechnology (NANOMED) at the University of Catania pays special attention to interdisciplinary research in the field of innovative treatments (drug delivery and targeting) and biomedical and pharmaceutical nanotechnologies [64].

The European Center of Personalized Medicine (ECPM) relies on scientific breakthroughs of personal molecular and genetic profiles, indicating what makes patients susceptible to certain diseases [65].

The Centre for Personalised Medicine (CPM) is a partnership between the University of Oxford’s Centre for Human Genetics and St Anne’s College, Oxford. It involves multidisciplinary communication, engagement, and scholarship to enable researchers, clinicians, academics, policy makers, and the public to explore the benefits and challenges of personalizing medicine, including clues about what needs to happen to enable effective introduction of scientific and technological advances in public health approaches and healthcare [66].

### 3.4. The Problems Related to Application of Molecular and Genetic Technologies

**Figure 2:**
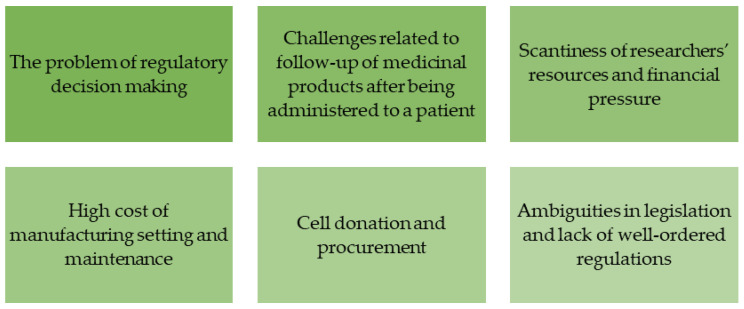
The problems related to application of molecular and genetic technologies.

Financial support to R&D makes companies engaged in it and the country as a whole superior in the high-tech market. In developed countries, the predominance of private sector investment into R&D indicates that companies form their own cradle-to-grave process flows (from development to the end of product manufacturing). These process flows are not built in all countries; so-called corporate science stays away from collaborations and focuses on its own productivity and demand, while basic research develops mostly autonomously and is not demanded by business corporations (Figure 2).

The problem of regulatory solutions or requirements in the application of high-tech gene therapy products is also relevant. The solutions to this problem should be aimed at introducing standards for assessment of the quality, safety, and efficacy of medicinal products, which would underlie regulations of the entire product’s life cycle. Regulatory compliance during the development of gene therapy and cell-based medicinal products, their manufacturing, and control will make it possible to assess the hallmarks of advanced therapies as well as the potential risks and severity of their sequelae. In particular, gene therapy medicinal products are regulated by separate legal frameworks for genetically modified organisms. The European Union has also established a regulatory system to control the compliance with this legislation [67,68,69]. However, adhering to these requirements when developing gene therapy medicinal products hampers their production [70].

The measures for following up the medicinal products after being administered to patients impede the implementation of gene therapy. Thus, in the European Union, the requirements are as follows:

follow-up of patients administered with gene therapy medicinal products must be at least 30 years;

traceability must be ensured, from the starting materials (e.g., donated material) to personal doses and, subsequently, patients [71].

The standards regulating safety and efficacy of gene therapy medicinal products (some of them formulated as part of the requirements for the manufacturing process and quality control) make these products very expensive. Because of limited resources, researchers are under permanent financial pressure. On the other hand, it stimulates them to search for the most cost-effective methods for developing and miniaturizing the production process.

The high cost of establishing and maintenance of manufacturing is also a limitation in the development of gene therapy and cell-based medicinal products because it needs to comply with strict requirements on the aseptic technique, ensuring constant environmental conditions, separating the manufacturing flows, etc. In the case of manufacturing of personalized medicinal products (e.g., for CAR-T cell therapy), the entire production cycle is performed to produce one dose for a single patient. There is a similar situation when a single dose or a relatively small quantity of the medicinal product needs to be obtained for quality assessment at different manufacturing stages.

Application of genetically modified organisms poses challenges related to cell donation and procurement, as well as risk assessment and reduction. For some medicinal products under development, there are no high-tech laboratories with an infrastructure enabling a wide range of tests using receptors, single cells, tissues, and large animals that would meet international requirements to ensure research data validity and humane use of animals.

The legislation in the field of genetic and cell-based developments is currently rather ambiguous and lacks order. The main problem related to regulations in this sphere is that there is no harmonization with international approaches, requirements, and definitions. Elaboration of special requirements imposed on manufacturing conditions and parameters, quality attributes being assessed, objectives, types, and scope of preclinical and clinical trials, as well as therapeutic use and risks for patients would significantly increase the efficiency of manufacturing gene and cell therapy products.

### 3.5. The Needs of the Development of Molecular and Genetic Technologies

Artificial cell systems

The key thesis of the cell theory proposed by M.J. Schleiden and T. Schwann in 1839 is that the cell is the fundamental unit of structure and function in all living organisms. However, studying only the structure, functions, and mechanisms of cell activity is no longer sufficient for modern cell biology.

There currently are several directions of cell research:

- cell engineering;
- bioengineering;
- medicine;
- targeted drug delivery;
- pharmaceutics;
- molecular biosensors;
- research into the origin of life, etc.

The interest in this field among the global community started to grow very recently, so timely support of the development of synthetic biology will allow Russia to compete with foreign researchers and make a technological breakthrough.

Genetically modified microorganisms are currently widely used in biotechnology. However, there are significant limitations to using genetically modified natural organisms to solve specific problems: living systems have an undefined number of unknown functions, which may yield unforeseen results and be a threat source. Furthermore, the features of organization of a living organism may not be suitable for solving specific tasks. Meanwhile, advanced technologies in synthetic biology are required even for the genome editing of living organisms. Modern gene therapy methods are based on targeted DNA delivery technologies. These technologies are both promising and dangerous, since their application can have both therapeutic effects and trigger the development of delayed-onset pathologies.

In addition, by mastering the technology of synthesizing long genomic sequences and then packing them into “containers” to ensure stability and self-reproduction, one can advance in creating binary genomes (a combination of viral, bacterial, and/or eukaryotic sequences). This approach can allow one to create and reproduce new organisms with tailored parameters and functions, which is a rather promising task for industrial technology, agriculture, and medicine; it can also be used for strengthening the country’s defense capability.

Searching for novel pathogens

The boosted level of technical capabilities and production capacity of research laboratories, biotechnological manufacturing facilities, as well as animal habitat alteration caused by climate change and land invasion by humans, contribute to the emergence of various biological threats.

Today, microorganisms with tailored properties can be created using modern biotechnology and genomic editing methods. Among these genetically modified organisms, there can be objects posing a threat to human health and, therefore, to a country’s biosecurity. Hence, timely identification of such modified microorganisms and assessment of the technical feasibility of creating them are the highest-priority tasks of laboratories that specialize in solving biological safety problems.

The risk of transmitting a new pathogen species to humans around the world is increasing due to global climate change. The success of preventing potential outbreaks of new diseases depends directly on how well the natural pathogen reservoirs, their intermediate hosts, and proximity to human habitats are known and characterized, as well as on the realistic assessment of potential events that may cause pathogen–human contact.

The common methods (such as PCR and ELISA) routinely used by clinical diagnostic laboratories allow simple and inexpensive detection of pathogens for which genome sequences are known, protein properties are well-studied, and the pathogenicity mechanisms have been analyzed.

Owing to the high-throughput sequencing methods, which have become commonly used over the past decade, and having conducted numerous diverse experiments, humanity has accumulated a large body of genetic information deposited in international or national databases. This information has partially been processed by authors and reported as publications. However, the speed of elaborating computational algorithms enabling the extraction of target information from the accumulated array of raw sequencing data expectedly lags behind the generation of this information (meanwhile, genetic information is being accumulated at a steadily increasing pace). Undoubtedly, the sequencing data for a broad range of biological and clinical samples that have already been obtained by different laboratories all over the world contain a large amount of implicit information about pathogens, and their identification is a relevant task that can be solved using bioinformatics methods. Therefore, elaboration of algorithms for detecting the genome sequences of pathogens using modern computational technologies is among the top priorities.

Microarrays

The advances in genomic and postgenomic research have provided great opportunities for understanding the functions of living organisms at the cellular and molecular levels. Companies specializing in developments in microelectronics and the pharmaceutical industry have started implementing the results of these studies in practical medicine at a remarkable pace, thus leading to establishment of molecular medicine.

Microarrays are characterized by multiplexity and high-speed performance; they are capable of real-time registration of a broad variety of markers of various pathologies, as well as a wide range of low-molecular-weight compounds (drug components, hormones, narcotic substances, etc.). Almost any biological material (plasma, serum, whole blood, sweat, saliva), water, soil samples, etc. can be used.

The future outlook of using microarrays is that there will no longer be a need for laboratories with their numerous staff members and expensive infrastructure, and analysis costs will be reduced.

Microarrays can be classified according to their manufacturing technology, type of material used, recognition receptor, and method employed for recording the results of interaction between the receptor and the analyzed biomolecule.

Today, there exist several types of microarrays:

- DNA microarrays (identification of genes and mutations in them, monitoring gene expression, diagnosing diseases and assessing therapy effectiveness, as well as screening of microorganisms);
- protein microarrays (identification and quantification of proteins during the development of various pathologies, assessment of the effectiveness of pharmaceuticals);
- cellular microarrays (analysis of interactions of protein molecules in the cell);
- tissue microarrays (analysis of tissue samples);
- small-molecule microarrays (one-step screening of a large number of potential drugs);
- microfluidic microarrays (repeated examination of biological objects).

RNA modifications

Today, it is clear that the central dogma of biology (“one gene–one protein”) is oversimplified. Because of alternative splicing, knowledge about the complete genome sequence does not mean that the proteome can be accurately identified. Although the term “epigenetics” was coined back in 1942, it was not until the past two decades that the importance of epigenetics regulation of gene expression has been recognized [72,73]. Other factors, including single-nucleotide polymorphisms (SNPs), polyploidy, and copy number variation, also need to be considered from the perspective of cell systems biology.

More than 20 types of RNAs in various organisms are currently known to be involved in gene regulation, posttranscriptional processing, DNA replication, and protein synthesis [74,75]. RNA modification is now an important factor in regulation of messenger RNA stability [27,76].

The systematic view of the cell

The choice of components that are actually analyzed in systems biology studies depends on research objectives and, to a greater extent, on the currently available technologies. Obviously, the lack of technological capabilities or knowledge required for performing systems biology analysis of the human cell should not be an obstacle for scientists striving for progress. In order to achieve it, cellular components need to be analyzed in a simplified manner; i.e., structural and functional assessment of only four major classes of biomolecules (DNA, RNA, proteins, and metabolites) needs to be performed. Each of these classes should further be subdivided into subclasses such as introns, exons, enhancers, promoters, etc. for DNA or lipids, metals, saccharides, etc. for metabolites. Specific types of information collected for each type of biomolecule can differ in modern systems biology. In genomics (DNAs), the main focus is placed on detection of mutations and the gene copy number; in transcriptomics (RNAs), the main focus is placed on the relative abundance and posttranscriptional modifications; while in proteomics (proteins), on the relative abundance of protein and the possible forms of postsynthetic modifications (PSMs); and in metabolomics, on quantity. Since any cell requires all four classes of these biomolecules to act in concert in order to function efficiently, the minimal information needed for assessing cell viability will include the data obtained in genomic, transcriptomic, proteomic, and metabolomic studies. In order to assess the interpretability of the view of the cell, one needs to delve into the types of data obtained for each class of biomolecule.

## 4. Conclusions

The evolution of the global personalized medicine market directly depends on innovations and developments in molecular and genetic technologies. It is worth noting that the existing problems in biomedicine and biotechnology cannot be solved by individual scientists. Activities stimulating progress in these fields of science should be carried out at an interdisciplinary level.

It is quite promising for evolution of the personalized medicine market to organize a good institutional environment throughout the entire “life cycle” of development and production of a biotechnological product, which can be ensured by: (1) government support and (2) large-scale investment in the acquisition of technologies and fabrication lines. It will also improve the scientific and technological capabilities of corporations and increase the demand for the research outputs. Furthermore, the process chains of large-scale industries should spread outside the country. Thus, during the COVID-19 pandemic, companies involved in cross-country cooperation succeeded in vaccine development and early market entry: BioNTech (Mainz, Germany) + Pfizer (State of New York, New York City, USA), Moderna (Cambridge, MA, USA) + Lonza (Basel, Switzerland).

Training of highly qualified personnel is also an efficient support measure for systems biology and biotechnology. Staff shortage is the major problem that is difficult to eliminate in a short- and even mid-term perspective. Geneticists, biotechnological engineers, biochemical engineers, specialists in biomedical analysis and visualization, systems and computational biologists, pharmaceutical microbiologists, etc. need to be recruited to develop the sphere of manufacturing of gene and cell products.

Different types of cooperation in the field of biotechnology can be crucial in the context of increasing R&D costs, the speed of response to external economic stress factors, and shortage of highly qualified personnel. Meanwhile, such factors as arrangement of rapid passing through the standardization and certification procedures, reduction of manufacturing and transportation costs, proximity of raw material markets, and the size of sales markets are very important.

State-of-the-art genetic engineering underlies biotechnology, which contributes to improvement of quality of human life and its expectancy, especially in terms of combating terminal illnesses [77]. Therefore, when refining the legal and regulatory framework for biotechnology application, one needs to take into account the systemic relationships with genetic technologies. For integrating the biological and genetic disciplines, the legal framework in these spheres needs to be mastered in the intersectoral context.

The technical challenges faced in drug manufacturing are solvable in the long run; however, there also exist problems related to research and legal regulations. Currently, the available advanced therapy medicinal products are typically used for treating rare diseases. In order to statistically confirm drug efficacy and/or safety, comparative clinical trials on a large sample (involving hundreds of individuals) need to be performed, which can be quite problematic for orphan diseases.

## Figures and Tables

**Table 1:** The search strategy summary.

Items	Specification
Databases and other sources searched	PubMed, Scopus, and Web of Science
Search terms used	“molecular technologies”, “genetic technologies”, “personalized medicine”, “biotechnology market”, “microarray”, “sequencing”, “omics technologies”, “mass spectrometry”, “gene therapy”, “RNA modifications”, “metabolomics”, “single cell”
Timeframe	2015–2024
Inclusion criteria	English language Peer-reviewed journals Full text
Included in analysis	77 manuscripts

**Table 2:** The list of biosensor-based devices and technologies designed in different geographic regions.

Product/Technology	Company	Characteristics	Reference
Medical device with reservoir-based sensors	MicroCHIPS, Inc. (Southfield, MI, USA)	Storing chemicals (reagents) and protecting them against environmental factors until the procedure of chemical reaction initiation and/or assay or biospecific recognition (sensing) is performed	United States Patent US8442611B2 [35]
DNA microarray based on a quartz ball (“geneball”)	University of Queensland (Brisbane, Australia)	The microarray surface contains channels 10 nm in diameter, with specific tagged DNA fragments. The microarray works like a barcode and aims to analyze human genetic information	[36]
Next-generation microarray based on the PHENOMICS^®^ technology	Proteus (Nîmes, France)	Ensures analysis of the proteome, genome, or its fragments	[37]
Biochip Array Technology	Randox Food Diagnostics (Crumlin, Co. Antrim, Ireland)	A biochip array sized 9 × 9 mm consisting of 44 test spots that enable screening of up to 48 food or fodder samples within less than 3 hrs	[38]
A1 Biochip	Archer Materials (Sydney, Australia)	Miniaturization of medical laboratory tests on the integrated circuit of a single array several millimeters in size. The array contains integrated microfluidic channels enabling gas or liquid sampling and miniaturized electrodes within the sensing regions made using gold and titanium metals	[39]
GeneChip	Affymetrix (Santa Clara, CA, USA)	The high-density array allows one to perform multiple independent measurements for each transcript call and genotype. The GeneChip system offers a comprehensive solution for elucidating the complex mechanisms and networks underlying the biological processes and diseases	[11]
SOI–NW biosensor	Institute of Biomedical Chemistry, Institute of Semiconductor Physics, Siberian Branch of the Russian Academy of Sciences (Moscow, Novosibirsk, Russia)	The microarrays forming this biosensor contain nano-sized silicon sensing elements. The NW biosensor is a molecular detector making it possible to register individual biological molecules in the counting mode, thus ensuring high concentration sensitivity of the analysis	[40]
Biosensor for simultaneous detection of glucose and lactate in blood	Moscow State University (Moscow, Russia)	A planar biosensor for detection of glucose and lactate in a liquid sample consists of a silver/silver chloride reference electrode and two working electrodes on a substrate, as well as a counter electrode placed between the working electrodes	[37]
A unique diagnostic platform	Akonni Biosystems, Inc. (Frederick, MD, USA)	The solution is based on the TruArray technology employing 3D gel microarrays for rapid simultaneous screening of the sample to detect hundreds of disease markers. The 3D gel droplet arrays have a sterically favorable distance between the immobilized molecules over the entire bulk of a gel droplet and the 99% aqueous environment surrounding the covalently linked probes	[41]
OciChip™ microarrays	Ocimum Biosolutions (Madhapur, India)	The process of microarray design and production involves two stages: (1) high-specificity and high-sensitivity microarrays; (2) oligonucleotides are manufactured and purified using strict quality control procedures	[42]
HOMIM	Foresight Institute (San Francisco, CA, USA)	The technology enables microbiological analysis of 300 species of the main bacteria that live in the oral cavity using 16S rRNA sequencing	[43]

**Table 3:** Examples of the application of nanotechnology in medicine.

Disease Groups	Description	Commercial Name	Ref.
Diagnostic tools and health monitoring			
Cancer	Hardware and software complex for multiplex immunoassay. Target molecules are caught on the surface of the sensor areas of the chip. Next, the target molecules are recognized by antibodies labeled with magnetic nanoparticles. Magnetic nanosensors detect the magnetic field formed by such “sandwich” immune complexes	MagArray (Milpitas, CA, USA)	[47]
Cancer, kidney diseases, bone defects, etc.	Gold nanoparticles as imaging contrast agents for X-ray/CT; gold nanoparticles with inclusion of heavy metals (gadolinium, iron oxide) for MRI; nanoparticles with radioisotopes (64Cu, 111In) for nuclear imaging. The size of particles can range from 1 nm to over 100 nm	AuroVist™ (Nanoprobes, Yaphank, NY, USA)	[48,49]
Cardiovascular diseases, neurological diseases, endoscopy	Nanogenerators are a class of self-powered and implantable medical nanosensors. Used for pacemakers, neurostimulators, and drug delivery systems	–	[50]
Medicines and vaccines			
Cancer	Iposomal injection doxorubicin hydrochloride—doxorubicin encapsulated in a closed lipid sphere	Doxil (San Francisco, CA, USA)	[51]
	Liposomal formulation of irinotecan, which is used in the treatment of malignant neoplasms	Onivyde (Roma, Italy)	[52]
	Albumin-bound 130 nm particle form of paclitaxel	Abraxane (Basking Ridge, NJ, USA)	[53,54]
Infectious diseases	Virosome-based adjuvanted influenza vaccine licensed for all age groups (from 6 months)	Inflexal V (Site Berne, Switzerland)	[55,56]
	Consists of formalin-inactivated hepatitis A virus (HAV)	Epaxal (Site Berne, Switzerland)	[55,56]

## Data Availability

No new data were created or analyzed in this study. Data sharing is not applicable to this article.
